# Investigating the causal links among gut microbiome features, inflammation-related proteins, and diverticular disease: Insights from a mediation Mendelian randomization study

**DOI:** 10.1097/MD.0000000000042676

**Published:** 2025-05-30

**Authors:** Jie Zhou, Yixin Xu, Haitao Wang, Kun Wang, Chao Chen

**Affiliations:** a Department of Gastrointestinal Surgery, The Wujin Hospital Affiliated with Jiangsu University, Changzhou, Jiangsu Province, China; b Department of Gastrointestinal Surgery, The Wujin Clinical College of Xuzhou Medical University, Changzhou, Jiangsu Province, China; c Department of Gastrointestinal Surgery, The Third Affiliated Hospital of Soochow University, Changzhou, Jiangsu Province, China.

**Keywords:** causal relationship, genetically predicted, GWAS, mediation analysis, single nucleotide polymorphism

## Abstract

The pathophysiological mechanisms underlying diverticular disease (DD) remain incompletely understood, and there is considerable debate regarding the roles of gut microbiome features and inflammation-related proteins in the development of the disease. In this study, we employed mediation Mendelian randomization (MR) analysis to investigate the causal relationships among these 3 factors. In this study, we conducted a MR analysis on the genome-wide association studies data of 412 gut microbiome features (207 microbial taxa and 205 pathways), 91 inflammation-related proteins, and DD. We employed the inverse-variance weighted (IVW) method as our primary screening approach, followed by a mediation MR analysis to explore potential causal relationships among these 3 aspects. Our findings were further reinforced by comprehensive heterogeneity analyses, horizontal pleiotropy testing, outlier detection, and “leave-one-out” sensitivity analysis. Through our screening process, we identified potential causal relationships between DD and 18 gut microbiome features, as well as 6 inflammation-related proteins. These include *s_Oscillibacter_unclassified* (IVW odds ratio (OR): 1.139; 95% confidence interval (CI): 1.044–1.241, *P* *=* .003), *g_Bilophila* (IVW OR: 1.107, 95% CI: 1.016–1.206, *P* *=* .020), T-cell surface glycoprotein CD5 levels (IVW OR: 1.065, 95% CI: 1.011–1.123, *P* *=* .019), and inosine 5’-phosphate biosynthesis I (IVW OR: 0.882, 95% CI: 0.800–0.973, *P* *=* .012), etc. In the mediation MR analysis, we found that the genetic predictors of *g_Bilophila* and inosine 5’-phosphate biosynthesis I could explain 23.956% and 24.630% of the variation in T-cell surface glycoprotein CD5 levels, respectively. This study detailed analysis of the links between gut microbiome features, inflammation-related proteins, and DD offers key insights into DD pathogenesis and prevention.

## 1. Introduction

Diverticular disease (DD) refers to the formation of outward pouches in the colon mucosal and submucosal layers.^[1]^ This condition typically occurs around penetrating blood vessels.^[2]^ The sigmoid colon is the most commonly affected area.^[3]^ Patients may experience abdominal pain, changes in bowel habits, rectal bleeding, and even serious complications such as diverticular perforation, which have a relatively high mortality rate.^[4,5]^ DD is wide spread globally, common in both Eastern and Western countries, with the highest reported incidence rates in the United States, Australia, and Western Europe.^[6]^

The pathophysiological mechanisms of DD remain incompletely understood.^[7]^ Various theories have been proposed regarding its etiology, encompassing abnormalities in the structure of the colon wall, disturbances in intestinal motility, low-fiber dietary habits, inflammation, and imbalances in the gut microbiota.^[8–10]^ Among these, the impact of a low-fiber diet is widely recognized.^[11]^ Importantly, the choices of diet and lifestyle are directly linked to the composition of the gut microbiome. Western dietary patterns and obesity are believed to be associated with reduced microbial diversity and changes in the microbial composition.^[12,13]^ Conversely, a high-fiber diet is thought to enhance the diversity of the gut microbiota^[14,15]^ and increase the production of short-chain fatty acids (SCFAs) by microbial species,^[16]^ which plays a crucial role in shaping immune function and strengthening the intestinal mucosal barrier.^[17]^ Given these findings, exploring the role of the gut microbiome features in the development of DD presents an intriguing area of research.

Regrettably, only a handful of studies have explored gut microbiota in the context of DD, and their findings are markedly contentious. Tursi et al identified an increased abundance of *Akkermansia muciniphila* in the fecal microbiota of DD patients compared to a healthy cohort.^[18]^ In contrast, Barbara et al observed lower levels of this bacterium in mucosal biopsies near diverticula compared to more distant areas in patients with symptomatic uncomplicated DD.^[19]^ Despite findings of reduced species like *Bacteroides fragilis* and *Collinsella* in DD patients’ feces,^[20]^ other studies found no significant differences in the microbial distribution between the colons of DD patients and healthy controls.^[21]^ These findings highlight the ongoing debate and inconsistency in understanding the relationship between DD and gut microbiota.

Inflammation plays a key role in the occurrence and recurrence of DD and diverticulitis.^[22]^ However, its role in the pathogenesis of DD remains unclear.^[23]^ For instance, research on tumor necrosis factor alpha levels in the colonic mucosa shows conflicting results: while some studies link elevated levels to the severity of DD,^[24]^ others find no correlation with its symptoms.^[25]^ Additionally, emerging evidence suggests that inflammation may play a role in the early pathogenic mechanisms of the disease,^[26]^ and its involvement in the etiology of DD could be more significant than previously thought.^[10]^

The intricate interactions among the gut microbiome features, inflammation, and DD continue to be elusive and are not yet fully elucidated. It is important to recognize that the current body of evidence establishing a connection between the gut microbiota, inflammatory processes, and DD is entirely based on observational studies. These studies are susceptible to potential confounding factors and may also involve reverse causality. Thus, there is a critical need to design and implement randomized research protocols to further elucidate this relationship.^[27]^ To overcome these challenges, employing Mendelian randomization (MR) analysis in conjunction with data from genome-wide association studies (GWAS) offers a promising strategy for investigating causal connections within assumed exposure–outcome pathways.^[28]^ MR capitalizes on the principle of the random allocation of genetic variants at conception, effectively mirroring a natural experiment. This method facilitates the exploration of possible causal links between risk factors and disease outcomes. It offers the distinct benefit of reducing the influence of confounding variables by leveraging their randomized distribution.^[29]^ In our research, we meticulously collected GWAS data for gut microbiome features, inflammation-related proteins, and DD for analysis. To the best of our knowledge, this represents the first attempt to use MR analysis to unravel the complex interactions among these 3 variables. This study is crucial for elucidating the pathogenesis of DD and identifying potential targets for future therapeutic strategies.

## 2. Methods

### 2.1. Study design

The data utilized in our analysis were publicly available and had been approved by the institutional review committees of the respective studies. Hence, no ethical committee review was necessary for this study. Furthermore, all findings generated are detailed in the article and its supplemental materials, Supplemental Digital Content, https://links.lww.com/MD/P83.

In this study, we explored the causal relationships between the gut microbiota, inflammation-related proteins, and DD through MR analysis. Additionally, we investigated whether there is a mediating effect among the 3. In our research, single nucleotide polymorphisms (SNPs) were defined as instrumental variables (IVs).^[30]^ Crucially, the integrity of MR analysis hinges on 3 fundamental tenets: (1) a demonstrable linkage between SNPs and the exposure variable; (2) an assurance that SNPs are not entangled with any confounders affecting the exposure–outcome relationship; and (3) a direct pathway through which SNPs influence the outcome exclusively via the exposure variable.^[31]^

### 2.2. GWAS summary data sources

The 412 gut microbiome features analyzed in our study were obtained from the Dutch Microbiome Project. This study conducted a comprehensive GWAS involving 7738 participants, covering 207 microbial taxa and 205 pathways, thereby representing microbial composition and function.^[32]^ It is important to emphasize that all GWAS data were meticulously gathered from various consortia or organizations, ensuring the absence of sample overlap (Table S1, Supplemental Digital Content, https://links.lww.com/MD/P83).

In addition, our study incorporates data on 91 inflammation-related proteins, which were derived from an extensive genomic analysis. This analysis utilized the Olink Target platform to measure plasma proteins in a large cohort of 14,824 individuals (Table S2, Supplemental Digital Content, https://links.lww.com/MD/P83). This was part of a protein quantitative trait locus study, specifically aimed at identifying the genetic determinants that modulate the expression of inflammation-related proteins.^[33]^

Lastly, the GWAS summary statistics for DD, which included 33,619 cases and 329,381 controls, were procured from the FinnGen consortium (Release 10). For detailed information on the aforementioned data, please refer to Table 1.

**Table 1:** Details of the genome-wide association studies and datasets used in our analyses.

Items	Sample size (cases/controls)	Data sources	PMID	Data download link
Diverticular disease	363000 (33,619/329,381)	FinnGen consortium	–	https://storage.googleapis.com/finngen-public-data-r10/summary_stats/finngen_R10_K11_DIVERTIC.gz
412 gut microbiome features (207 microbial taxa and 205 pathways)	7738	DMP	35115690	https://www.ebi.ac.uk/gwas/; Accession numbers GCST90027446-GCST90027857
91 inflammatory-related proteins	1,4824	Nature immunology	37563310	https://www.ebi.ac.uk/gwas/; Accession numbers GCST90274758 to GCST90274848

DMP = Dutch Microbiome Project.

### 2.3. IVs selection and data harmonization

In our investigation, we adhered to rigorous selection protocols for SNPs to fortify the robustness of our findings. Drawing on the methodologies of previous studies,^[34,35]^ we focused on SNPs that met the threshold for genome-wide significance (*P* *<* 1 × 10^-5^) for intensive examination. To maintain the integrity of our IVs, we meticulously removed any palindromic and ambiguous SNPs.^[36]^ We then grouped SNPs based on linkage disequilibrium within a 10,000 kb radius, applying an *r*^2^ cutoff of <0.001.

The F-statistic was utilized as an indicator to gauge the variance explained by each SNP, calculated using the formula [(N - K - 1)/K]/ [*R*^2^/(1 - *R*^2^)], where “K” denotes the number of genetic instruments and “N” the sample size. IVs that yielded an F-statistic below 10 were deemed insufficiently robust and were thus excluded to enhance the precision of the analysis.^[37]^

Additionally, we undertook a thorough literature review to scrutinize all phenotypes associated with the genetic instruments in our study. This step was crucial to exclude any SNPs potentially linked to confounding variables, thereby safeguarding the validity of our causal inferences.

### 2.4. Part one: the causal relationship between 412 gut microbiome features and DD

To investigate the causal relationships between 412 gut microbiome features (207 taxonomies and 205 pathways) and DD, we conducted MR analyses using GWAS data for gut microbiome features as the exposure and DD as the outcome. The cornerstone of our analytical strategy in this study is the IVW method, which integrates meta-analytic techniques with individual SNP Wald estimates. Under the assumption that horizontal pleiotropy is absent, the IVW method is poised to yield unbiased estimates, with significance demarcated at *P* < .05.^[38]^

To bolster this primary analysis, we engaged supplementary methods such as Bayesian weighted MR^[39]^ and the weighted median method.^[40]^ Notably, the Bayesian weighted MR method is designed for nuanced causal inference, proficiently addressing the challenges posed by minor effects in polygenic attributes. It leverages Bayesian weighting to discern outliers and to attenuate the effects of violations in the pleiotropic IV assumptions. The weighted median strategy, by offering a more constrained standard deviation relative to the MR-Egger technique, augments the preciseness of our estimates. It is particularly commendable for its ability to deliver dependable results even in the presence of horizontal pleiotropy, tolerating up to 50% of genetic variation stemming from potentially invalid instruments.^[41]^

### 2.5. Part 2: the causal links between 91 inflammation-related proteins and DD

To elucidate the causal relationship between 91 inflammation-related proteins and DD, we carried out two-sample MR analyses utilizing GWAS data for inflammation-related proteins as the exposure and DD as the outcome. Similarly, we employed the IVW method for systematic screening.

### 2.6. Reverse MR analysis

To determine whether there exists a reverse causal relationship between DD and the 2 exposures–gut microbiome features and inflammation-related proteins, we conducted reverse MR analyses. In these analyses, GWAS data for DD was used as the exposure, with the screened microbial features and inflammation-related proteins serving as the outcomes. The IVW method was again our analytical tool of choice. Any associations with a *P*-value < .05 were excluded from further consideration in our study.

### 2.7. Mediation analysis

**Figure 1.**
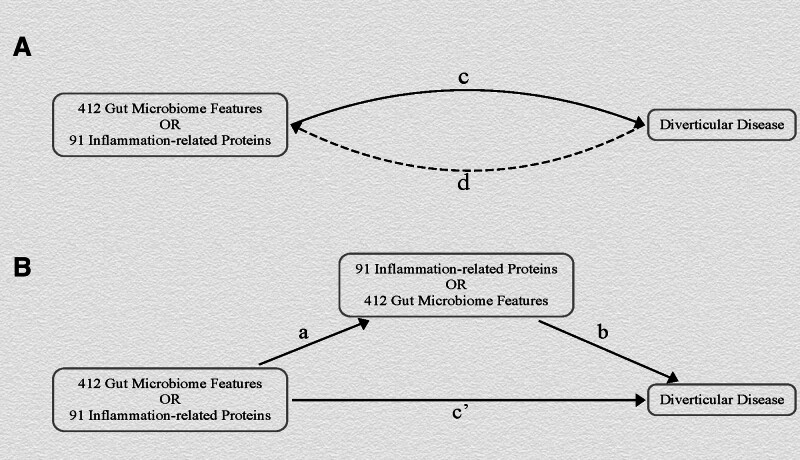
Diagrams illustrating associations examined in this study. (A) The total effect of gut microbiome features and inflammation-related proteins on diverticular disease (DD). “*c*” represents the total effect when using genetically predicted gut microbiome features and inflammation-related proteins as the exposure, with DD as the outcome. “*d*” denotes the total effect when using genetically predicted DD as the exposure, and gut microbiome features and inflammation-related proteins as the outcome. (B) The total effect is decomposed into: (i) the indirect effect using the two-step method (where “*a*” represents the effect between gut microbiome features and inflammation-related proteins, and “*b*” is the effect of the mediator on DD), and the product of method (*a* × *b*); (ii) the direct effect (*c*′ = *c* - *a* × b). The mediation proportion is the indirect effect divided by the total effect.

To explore whether gut microbiome features and inflammation-related proteins serve as mediators in the development of DD, we conducted mediation analyses with each of these factors acting as mediators (as shown in Fig. 1B). The total effect (denoted as *c* in Fig. 1A) can be decomposed into an indirect effect (*a* × *b* in Fig. 1B) and a direct effect (*c′* in Fig. 1B).^[42]^ We quantified the proportion mediated by calculating the ratio of the indirect effect to the total effect.

### 2.8. Sensitivity analysis

To reinforce the credibility of our results, we adopted several methodological strategies. Initially, we addressed potential heterogeneity in our two-sample MR analysis, which could arise from variations in experimental designs, study demographics, and SNPs, potentially distorting the estimation of causal relationships. To manage this, we employed both the IVW and MR-Egger methods to assess heterogeneity. Cochrane Q statistic served to quantify the heterogeneity among our genetic instruments, with a *P*-value > .05 signifying no significant heterogeneity.^[43]^

Moreover, a fundamental assumption of MR analysis is that the IV influences the outcome exclusively through the exposure, underscoring the necessity to examine potential horizontal pleiotropy that might confound the exposure–outcome relationship.^[44]^ We utilized the MR-Egger intercept method to probe for pleiotropy, where a *P*-value > .05 indicated minimal or no significant pleiotropic effects, thereby validating the robustness of our causal inference.

Additionally, within the IVW analysis, we employed the MR-PRESSO test to identify outliers, and subsequently, we refined our analysis after excluding these outliers.^[45]^ Lastly, we performed a “leave-one-out” analysis to ascertain the influence of individual SNPs on the exposure–outcome relationship, further strengthening the reliability of our findings.^[46]^

### 2.9. Statistical analysis

We conducted MR analysis using R software (version 4.2.0, https://www.r-project.org) in conjunction with the “Two-Sample MR” package (version 0.5.6) for precise and comprehensive analysis.

## 3. Results

**Figure 2.**
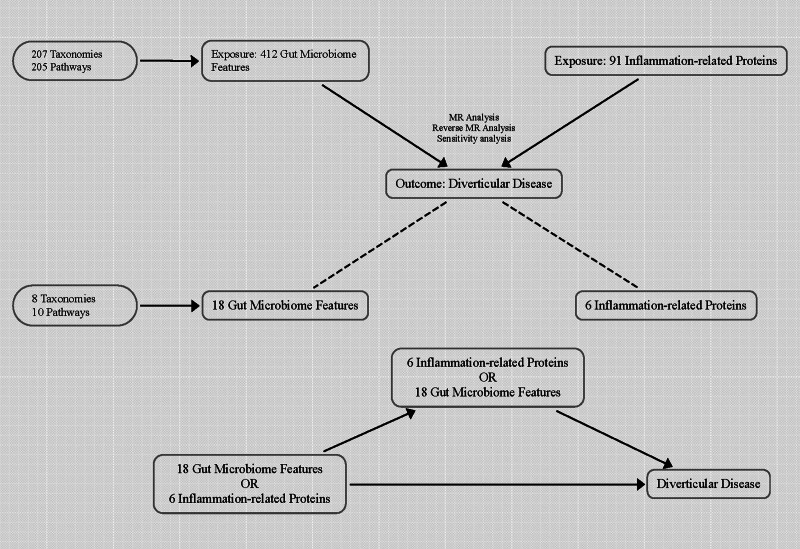
The workflow diagram for this study. IVW = inverse-variance weighted; MR = Mendelian randomization.

The workflow diagram for this study was shown in Figure 2.

### 3.1. Association of 412 gut microbiome features and DD

To elucidate the genetically predicted causal relationships between gut microbiome features and DD, we evaluated 412 such features as potential exposures, with DD as the outcome. Employing the IVW method for our preliminary screening, we identified 23 gut microbiome features that exhibited potential causal associations with DD.

Subsequent outlier detection within our MR analyses flagged anomalies specifically in the data concerning *p_Actinobacteria* and *c_Actinobacteria*. Upon the exclusion of these outliers, the correlations with DD failed to reach statistical significance, necessitating their removal from further consideration. Moreover, all but one of the remaining features, *GLYCOCAT.PWY: glycogen degradation I (bacterial*), excluded due to horizontal pleiotropy, demonstrated robustness in tests for heterogeneity, horizontal pleiotropy, and “leave-one-out” sensitivity analysis (Table S7, Supplemental Digital Content, https://links.lww.com/MD/P83).

**Figure 3.**
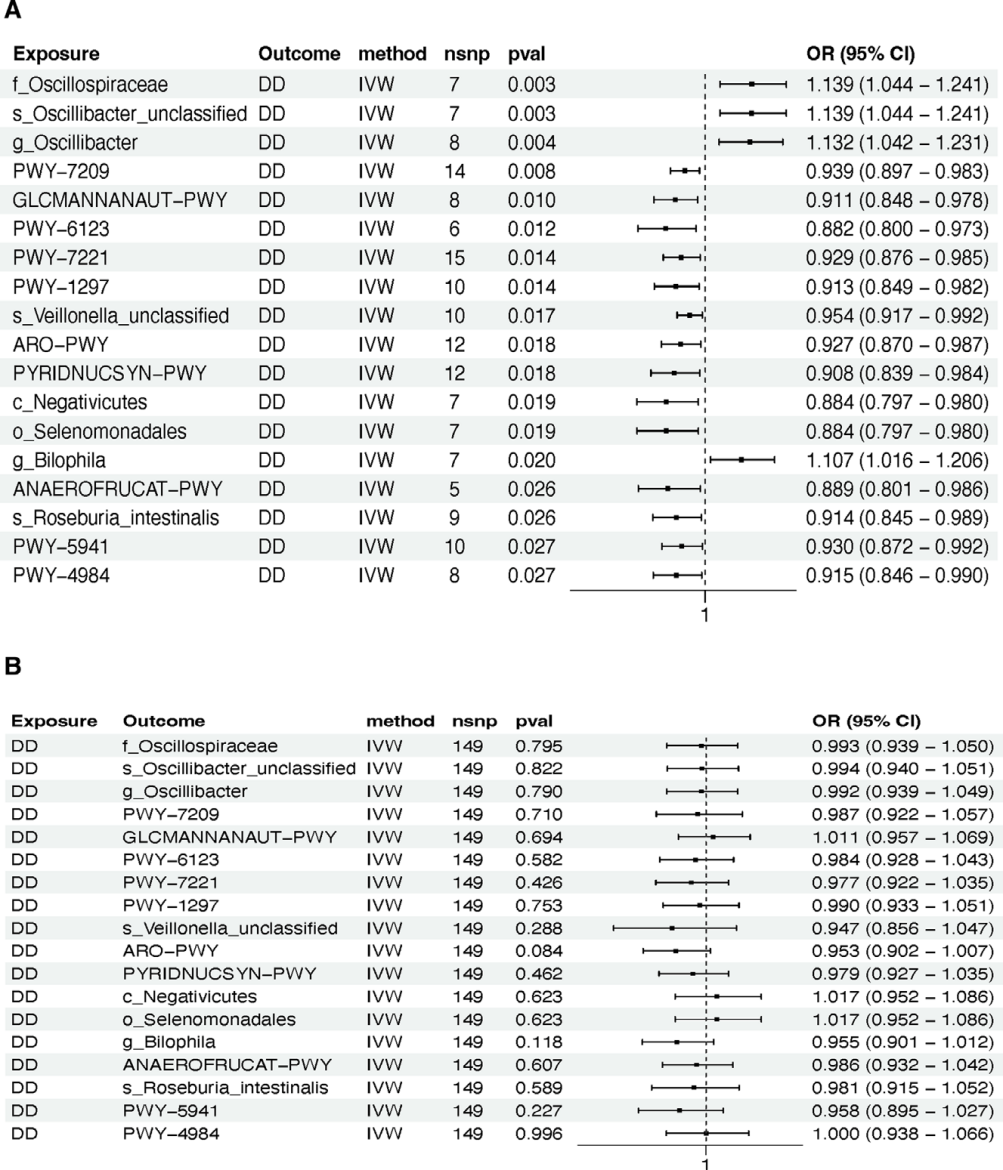
Use a forest plot to visually display the causal relationships between 18 gut microbiome features and DD. (A) Results of the IVW method in MR analysis; (B) results of the IVW method in reverse MR analysis. ANAEROFRUCAT-PWY = the pathway for anaerobic fructoselysine degradation, ARO-PWY = chorismate biosynthesis I, CI = confidence interval, DD = diverticular disease, GLCMANNANAUT-PWY = the superpathway of N-acetylglucosamine, IVW = inverse-variance weighted, MR = Mendelian randomization, OR = odds ratio, PWY-7209 = superpathway of pyrimidine ribonucleosides degradation, PWY-6123 = inosine 5’-phosphate biosynthesis I, PWY-7221 = guanosine ribonucleotides de novo biosynthesis, PWY-1297 = superpathway of purine deoxyribonucleoside degradation, PYRIDNUCSYN-PWY = NAD biosynthesis I from aspartate, PWY-5941 = glycogen degradation II (eukaryotic), PWY-4984 = urea cycle, SNP = single nucleotide polymorphism.

In an effort to preclude the possibility of reverse causation, reverse MR analyses were also conducted, positioning DD as the exposure and the gut microbiome features as the outcomes. This revealed reverse causal relationships for *g_Phascolarctobacterium* and *s_Phascolarctobacterium_succinatutens* with DD, resulting in their exclusion from the study. In the final assessment, 18 gut microbiome features, encompassing 8 taxonomic classifications and 10 metabolic pathways, met our stringent inclusion criteria and were thus selected for subsequent research (Table S3, Supplemental Digital Content, https://links.lww.com/MD/P83) (Fig. 3A and B). The characteristics of SNPs associated with 18 gut microbiome features and DD in the bidirectional MR analysis are extensively documented in the supplementary files (Table S4, Supplemental Digital Content, https://links.lww.com/MD/P83).

### 3.2. Association of 91 inflammation-related proteins and DD

To investigate the causal relationship between inflammation-related proteins and DD, we conducted a two-sample bidirectional MR analysis, treating 91 inflammation-related proteins as exposures and DD as the outcome. Utilizing the IVW method for initial screening, we identified 8 inflammation-related proteins with potential causal links to DD.

During subsequent outlier detection, we identified outliers in the MR analysis of tumor necrosis factor ligand superfamily member 12 levels with DD. After excluding these outliers and reanalyzing the data, we observed significant heterogeneity in the results, leading to the exclusion of this protein from further study. In the “leave-one-out” sensitivity analysis, the single genetic IV (rs9469127) for C-C Motif Chemokine 19 levels had a substantial influence on the exposure–outcome relationship. After excluding this IV and reanalyzing the data, the results did not reach statistical significance, leading to its exclusion as well (Table S7, Supplemental Digital Content, https://links.lww.com/MD/P83).

**Figure 4.**
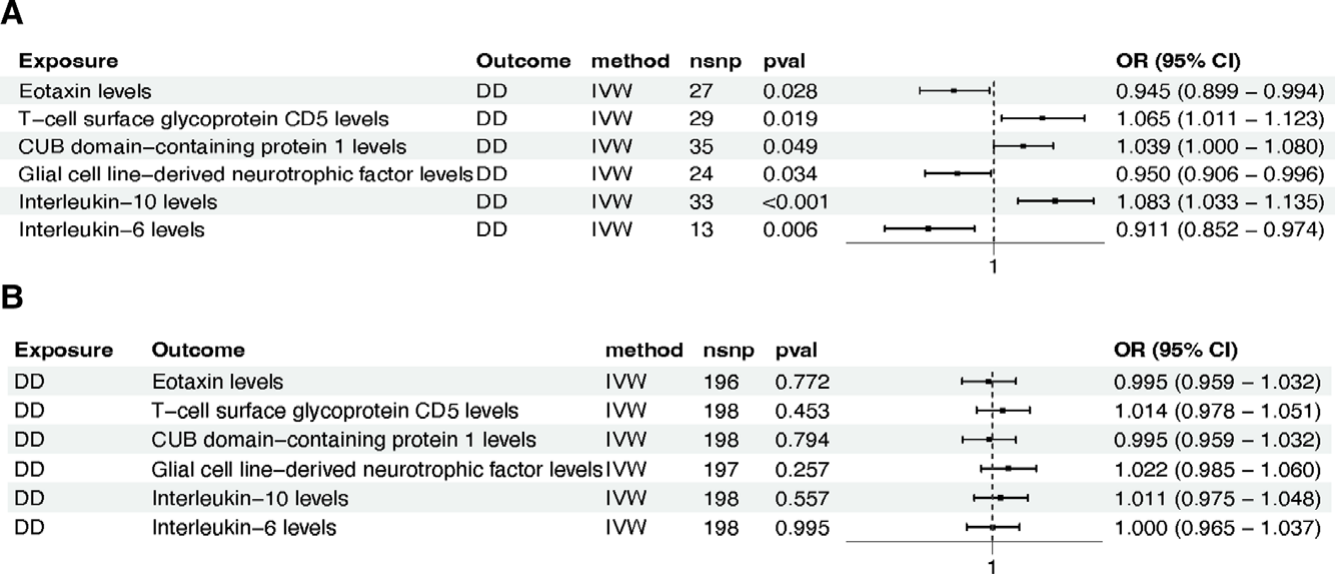
Use a forest plot to visually display the causal relationships between 6 inflammation-related proteins and DD. (A) Results of the IVW method in MR analysis; (B) results of the IVW method in reverse MR analysis. CI = confidence interval, DD = diverticular disease, IVW = inverse-variance weighted, MR = Mendelian randomization, OR = odds ratio, SNP = single nucleotide polymorphism.

To further explore potential reverse causality between the aforementioned inflammation-related proteins and DD, we conducted reverse MR analysis, treating DD as the exposure and inflammation-related proteins as the outcomes. The analysis revealed no reverse causal relationships. In conclusion, 6 inflammation-related proteins met the study criteria and were included in subsequent research (Table S5, Supplemental Digital Content, https://links.lww.com/MD/P83) (Fig. 4A and B). The characteristics of SNPs associated with 6 inflammation-related proteins and DD in the bidirectional MR analysis are extensively documented in the supplementary files (Table S6, Supplemental Digital Content, https://links.lww.com/MD/P83).

### 3.3. Investigation of the mediating mechanisms of inflammatory-related proteins

Through our analysis, we have identified 18 gut microbiome features and 6 inflammation-related proteins that may have a causal relationship with DD. To explore whether inflammation-related proteins serve as mediators in the link between gut microbiome features and DD, we conducted a mediation MR analysis.

**Figure 5.**
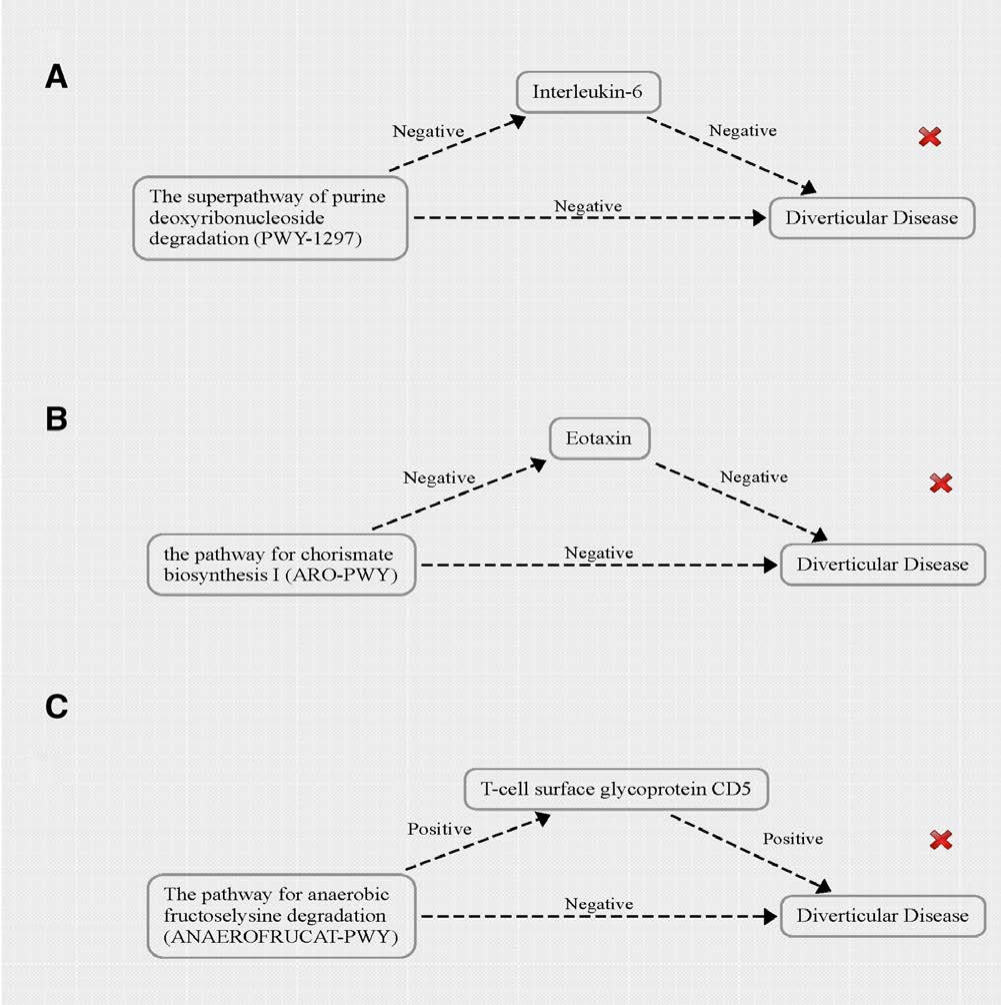
The causal relationship between gut microbiome features and diverticular disease mediated by inflammation-related proteins.

Overall, the superpathway of purine deoxyribonucleoside degradation (PWY-1297) has a negative correlation with DD. Moreover, PWY-1297 is negatively associated with interleukin-6 levels (IL-6), which in turn are also negatively correlated with DD (Fig. 5A). In addition, the pathway for chorismate biosynthesis I (ARO-PWY) shows a negative relationship with DD, and ARO-PWY is negatively correlated with Eotaxin levels, which are also negatively associated with DD (Fig. 5B). Finally, the pathway for anaerobic fructoselysine degradation (ANAEROFRUCAT-PWY) is negatively related to DD, while ANAEROFRUCAT-PWY has a positive correlation with the levels of T-cell surface glycoprotein CD5, which is also positively associated with DD (Fig. 5C). Consequently, none of the aforementioned pathways satisfies the criteria for mediation in MR analysis.

### 3.4. Investigation of the mediating mechanisms of gut microbiome features

To explore whether gut microbiome features serve as mediators in the link between inflammation-related proteins and DD, we conducted another mediation MR analysis.

**Figure 6.**
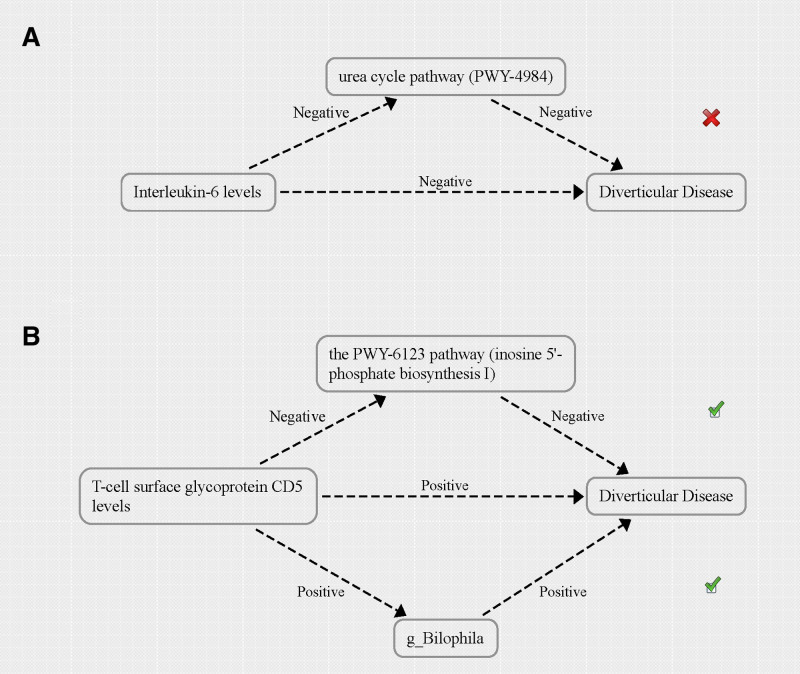
The causal relationship between inflammation-related proteins and diverticular disease mediated by gut microbiome features.

In summary, there is a negative correlation between IL-6 levels and DD. Furthermore, IL-6 levels are negatively associated with the urea cycle pathway, which in turn is also negatively related to DD (Fig. 6A). The causal relationships among these factors do not meet the mediation criteria in MR analysis.

In our study, it is noteworthy that the causal relationship between T-cell surface glycoprotein CD5 levels and DD appears to be mediated by 2 factors: the PWY-6123 pathway (inosine 5’-phosphate biosynthesis I) and the bacterium *g_Bilophila*. Specifically, there is a positive causal relationship between T-cell surface glycoprotein CD5 levels and DD. On one hand, there is a positive correlation between T-cell surface glycoprotein CD5 levels and *g_Bilophila* levels, with *g_Bilophila* also positively associated with DD. On the other hand, a negative correlation exists between T-cell surface glycoprotein CD5 levels and the PWY-6123 pathway, which in turn is negatively associated with DD (Fig. 6B). Both mediators met the criteria for conducting mediated MR analysis.

#### 3.4.1. Proportion of the association between T-cell surface glycoprotein CD5 levels and DD mediated by *g_Bilophila*

**Figure 7.**
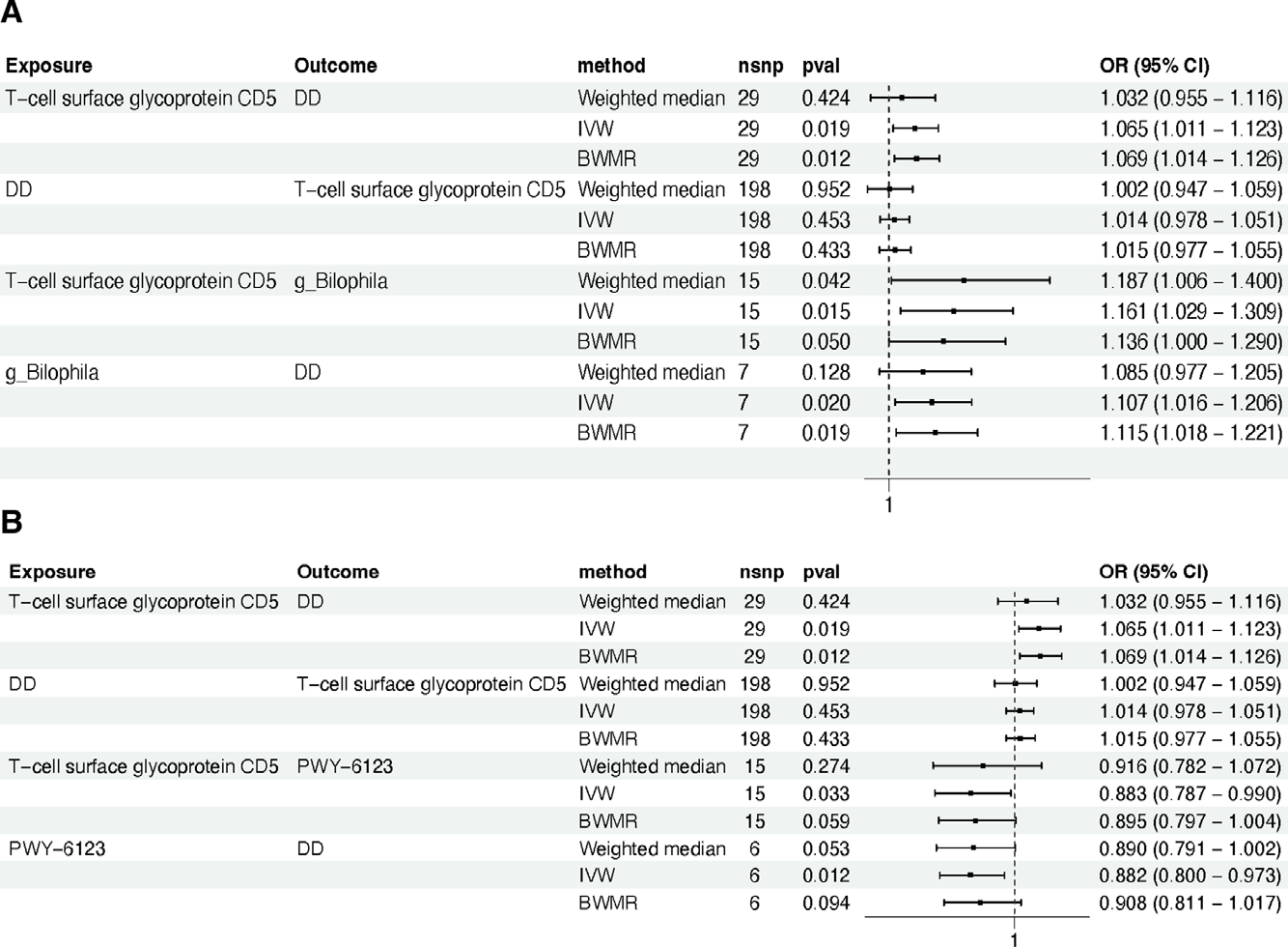
Utilize forest-plots to visually illustrate the causal relationships mediated by mediating factors. (A) The causal link between T-cell surface glycoprotein CD5 levels and DD, mediated by *g_Bilophila*. (B) The causal relationship between T-cell surface glycoprotein CD5 levels and DD, mediated by the PWY-6123 pathway. CI = confidence interval, DD = diverticular disease, OR = odds ratio, PWY-6123 = inosine 5’-phosphate biosynthesis I.

**Figure 8.**
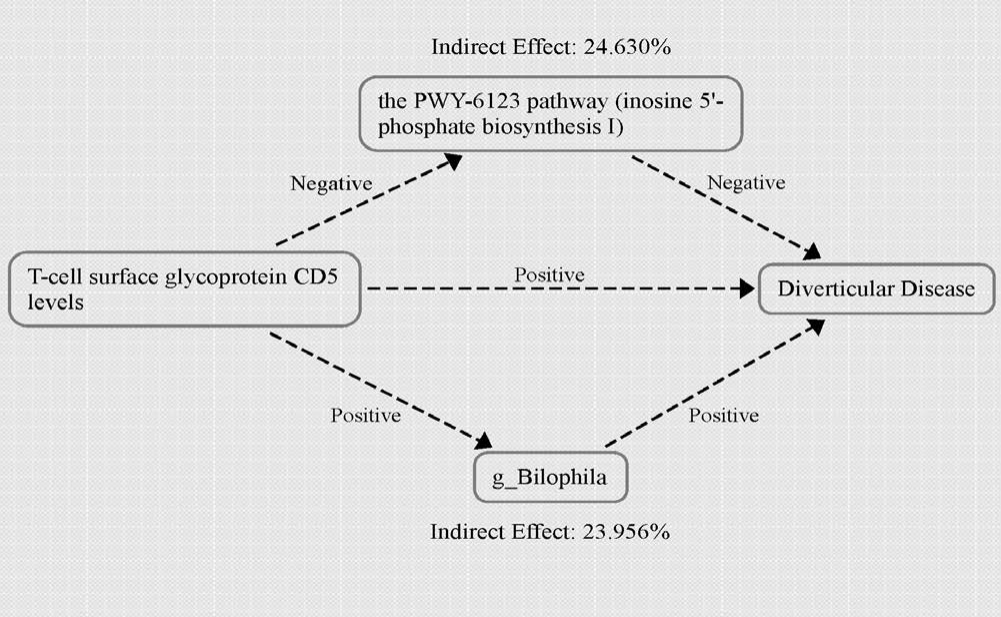
Schematic diagram of the mediation effect of gut microbiome features.

In summary, our research has conclusively identified *g_Bilophila* as a mediating factor in the causal pathway linking T-cell surface glycoprotein CD5 levels to DD. We found a significant association between higher levels of T-cell surface glycoprotein CD5 and increased abundance of *g_Bilophila*, which was subsequently associated with an increased risk of DD (Fig. 7A). In all the analyses mentioned above, no outliers, pleiotropy, or heterogeneity were detected, and they all meet the requirements of the “leave-one-out” sensitivity analysis (Table S7, Supplemental Digital Content, https://links.lww.com/MD/P83; Figure S1, Supplemental Digital Content, https://links.lww.com/MD/P84). As depicted in Figure 8, our analysis revealed that the indirect effect mediated by *g_Bilophila* was 0.015, accounting for 23.956% of the total increased risk of DD attributable to elevated levels of T-cell surface glycoprotein CD5. The direct effect, independent of *g_Bilophila*, was quantified at 0.048.

#### 3.4.2. Proportion of the association between T-cell surface glycoprotein CD5 levels and DD mediated by PWY-6123 pathway

Furthermore, our study has also identified the PWY-6123 pathway as a mediating factor in the causal relationship between the levels of T-cell surface glycoprotein CD5 and DD. We observed a significant negative correlation, where an increase in T-cell surface glycoprotein CD5 levels was associated with a decrease in the activity of the PWY-6123 pathway. Intriguingly, a reduction in the PWY-6123 pathway activity was linked to an elevated risk of DD (Fig. 7B). In all the analyses mentioned above, no outliers, pleiotropy, or heterogeneity were detected, and they all meet the requirements of the “leave-one-out” sensitivity analysis (Table S7, Supplemental Digital Content, https://links.lww.com/MD/P83; Figure S2, Supplemental Digital Content, https://links.lww.com/MD/P84). As illustrated in Figure 8, our analysis demonstrated that the indirect effect mediated by the PWY-6123 pathway amounted to 0.016, representing 24.630% of the total increase in DD risk attributed to the elevation of T-cell surface glycoprotein CD5 levels. The direct effect, independent of the influence of PWY-6123 pathway, was quantified at 0.048.

## 4. Discussion

DD is prevalent worldwide, with notably high incidence rates in the United States, Australia, and Western Europe.^[6]^ Despite its prevalence, the pathophysiological mechanisms underlying DD remain poorly understood.^[9]^ It is widely speculated that the gut microbiota and inflammatory responses may play significant roles in the formation and progression of DD, yet this area remains contentious. To our knowledge, our study is the first to employ MR to identify potential causal relationships between 18 gut microbiome features, 6 inflammation-related proteins, and DD. Despite inconsistencies in previous studies regarding the role of *Akkermansia muciniphila* in the development of DD,^[18,19]^ our MR analysis did not find a causal relationship between them. We speculate that observational studies may be prone to biases due to confounding factors, or that changes in this bacterial population could be a delayed response to the progression of DD. Importantly, through the use of mediation MR analysis, we discovered that the PWY-6123 pathway and *g_Bilophila* can mediate the effects of T-cell surface glycoprotein CD5 on DD. Notably, all our MR analyses satisfied the requirements for heterogeneity tests, horizontal pleiotropy tests, outlier analysis, and “leave-one-out” sensitivity analysis, ensuring the reliability of our findings.

In our MR analysis of gut microbiome features and the risk of DD, we identified a potential causal relationship between DD risk and 3 categories: *f_Oscillospiraceae*, *s_Oscillibacter_unclassified*, and *g_Oscillibacter*. We observed that these categories are interconnected within the hierarchical structure of bacterial classification. This suggests that *Oscillibacter* may play a significant role in promoting the onset and progression of DD. Li et al discovered that in mice treated with dextran sulfate sodium, the abundance of *Oscillibacter* in the gut increased, potentially leading to more severe inflammatory responses in the intestine.^[47,48]^ Studies have shown that *Oscillibacter* is associated with increased intestinal permeability.^[49]^ Moreover, the abundance of *Oscillibacter* significantly rises in mice fed a high-fat diet.^[50]^ Interestingly, a low-fiber diet is considered one of the key factors contributing to the development of DD.^[8]^ Therefore, we hypothesize that a low-fiber diet may influence the abundance of *Oscillibacter* in the gut, leading to the production of inflammatory metabolites and exacerbating inflammatory responses,^[51]^ thereby promoting the development of DD. Furthermore, this study has discovered that *c_Negativicutes* and its subordinate classification, *c_Negativicutes.o_Selenomonadales*, exhibit protective effects against the development of DD. The comprehensive analysis suggests that these 2 classifications may represent different hierarchical levels of the same species. Research indicates that the *Selenomonadales*, belonging to the phylum Firmicutes, make a significant contribution to the human gut microbiota and produce SCFAs.^[52]^ Numerous studies have found that SCFAs in the gut can enhance microbial diversity, and SCFAs have a significant impact on the host immune system and mucosal barrier.^[15–17]^ Additionally, we discovered that the s_*Veillonella_unclassified* acts as a protective factor in inhibiting the occurrence of DD. *Veillonella*, a gram-negative anaerobic coccus, is commonly identified alongside lactobacilli as part of the ileum microbiota.^[53]^ Studies have indicated that the production of acetate and propionate by *Veillonella* could benefit both the host and other symbiotic bacteria.^[54,55]^ *Veillonella* plays a significant role in the human microbiome, infection responses, and the development of immunity.^[34]^ For instance, in children prone to asthma, the abundance of *Veillonella* in the gut is significantly reduced.^[56]^ However, the potential protective effects of *Veillonella* in preventing or treating DD remain to be further explored. Moreover, our research has identified *g_Bilophila* as a potential risk factor that may lead to the development of DD. Studies have shown that a high-fiber diet can effectively reduce the abundance of harmful bacteria such as *g_Bilophila*.^[57]^ As a type of sulfate-reducing bacteria, *Bilophila* can produce hydrogen sulfide through dissimilatory sulfate reduction, a chemical compound that is highly toxic to all living organisms.^[58,59]^ Not only can hydrogen sulfide directly trigger intestinal inflammation, but it can also inhibit the metabolism of butyrate.^[60]^ The biofilms formed by sulfate-reducing bacterias in the gut can penetrate the vascular wall in cases of intestinal epithelial damage, increasing the risk of inflammation.^[61]^ These findings underscore the importance of modulating the gut microbiome composition, particularly in reducing the abundance of *g_Bilophila*, for the prevention and treatment of DD. Finally, we discovered a negative causal relationship between *s_Roseburia_intestinalis* and the risk of DD. Butyrate, a SCFA produced by *Roseburia*, has been shown to improve colonic inflammation.^[62]^ Numerous studies have found that SCFAs in the gut can enhance microbial diversity and have significant effects on the host immune system and mucosal barrier.^[15,16]^ Additionally, *Roseburia* interacts with plant polysaccharides to inhibit glycolysis and promote fatty acid utilization.^[63]^ Its anti-inflammatory effects and mechanisms in regulating mucosal immunity in patients with inflammatory bowel disease have also been extensively studied.^[64,65]^ Consequently, *Roseburia_intestinalis* is regarded as a potentially beneficial microbe, and its role in DD warrants further investigation.

In addition to an in-depth examination of the gut microbiome, our research has analyzed a multitude of bacterial metabolic pathways for their causal relationship with DD. Through rigorous screening, we identified a negative correlation with the following pathways: the superpathway of pyrimidine ribonucleosides degradation, the superpathway of N-acetylglucosamine, PWY-6123, guanosine ribonucleotides de novo biosynthesis, PWY-1297, ARO-PWY, NAD biosynthesis I from aspartate, ANAEROFRUCAT-PWY, glycogen degradation II (eukaryotic), and the urea cycle. This suggests that activation of these pathways may contribute to a reduced incidence of DD. We further discovered that these bacterial pathways are primarily involved in nucleotide metabolism, carbohydrate metabolism, energy metabolism, nitrogen and amino acid metabolism, and the biosynthesis of aromatic compounds. However, the exact mechanisms underlying these relationships require further investigation. These findings offer new insights into the metabolic underpinnings of DD and suggest novel avenues for the development of potential prevention and treatment strategies. These advancements promise to propel further research into DD and have the potential to lead to more effective therapeutic options for patients.

Although inflammation is a hallmark of acute diverticulitis and its complications, the role of inflammation or immune activation in the formation of diverticula or the manifestation of symptoms in patients remains a topic of debate.^[9]^ In this study, we identified 6 inflammation-related proteins that may have a causal relationship with the onset and progression of DD. Specifically, the genetic predisposition to elevated levels of T-cell surface glycoprotein CD5, CUB domain-containing protein 1, and Interleukin-10 (IL-10) has been associated with an increased risk of developing DD. This suggests that these inflammation-related proteins may play a promotive role in the pathogenesis of DD. Conversely, elevated levels of eotaxin, glial cell line-derived neurotrophic factor, and IL-6 have been found to potentially exert a protective effect in the disease process of DD. These findings highlight the multifaceted roles that different proteins play in the development and progression of DD. Currently, there is considerable controversy regarding the role of inflammation-related proteins in DD. An increase in T-cell surface glycoprotein CD5 levels often represents the activation or increase of T cells. In certain inflammatory responses or immune reactions, T cells are activated, leading to an elevation in the expression levels of CD5. Some studies have shown that the number of T cells, IL-10, tumor necrosis factor alpha, and neutrophils in the colonic mucosa of DD patients is significantly higher than that in healthy individuals.^[66–68]^ These inflammation-related proteins participate in various cellular processes and play a role in the regulation of inflammatory pathways, such as inducible nitric oxide synthase and cyclooxygenase-2.^[69]^ However, other studies have indicated that there are no significant differences in the levels of T lymphocytes, IL-10, IL-6, and C-reactive protein in the colonic mucosa between DD patients and the healthy population.^[21,25,70]^ Our research results may offer a new perspective on this controversy, but a limitation is that our samples of inflammation-related proteins were taken from plasma, whereas previous studies were mainly focused on the intestinal mucosa.

It is noteworthy that our study uncovered a causal relationship between the levels of T-cell surface glycoprotein CD5 and DD, with a minor part of this effect being mediated through *g_Bilophila* and the biosynthesis pathway of inosine 5’-phosphate I. Furthermore, in our discussions, we have thoroughly explored the potential causal mechanisms and the supporting research connecting the composition of the gut microbiome features, inflammation-related proteins, and DD. Although we have linked these elements together through mediation MR analysis, further research is required to validate these findings.

Our analysis presents both strengths and limitations of the study. Firstly, to the best of our knowledge, this research is the first to employ MR to conduct a comprehensive analysis of numerous gut microbiome features, inflammation-related proteins, and DD, with the aim of resolving disputes in this field. Our study ultimately identified 18 gut microbiome features and 6 inflammation-related proteins that play a significant role in the pathogenesis of DD, with a particular emphasis on the impact of gut microbiota. Secondly, through the application of mediation MR analysis, we were able to establish a link between these 3 factors. We discovered a causal relationship between the levels of T-cell surface glycoprotein CD5 and DD, with a minor portion of this effect being mediated through *g_Bilophila* and the biosynthesis pathway of inosine 5’-phosphate I. This finding holds significant implications for gaining a deeper understanding of the pathophysiology of diverticula. However, our study also bears certain limitations. Firstly, as the genetic analysis was primarily targeted at European patients, this may limit the generalizability of our findings to other ethnicities. Secondly, the adoption of a lower significance threshold for SNP selection could potentially impact the reliability of our results. Lastly, although we linked inflammation-related proteins, gut microbiome features, and DD through mediation MR analysis, the relative scarcity of research in this area suggests that the specific mechanisms of action may need further exploration and validation in future studies.

## 5. Conclusion

In this study, we conducted a MR analysis on the GWAS data of 412 gut microbiome features, 91 inflammation-related proteins, and DD. Ultimately, we identified 18 gut microbiome features and 6 inflammation-related proteins that may have a causal relationship with the onset and progression of DD. Furthermore, through mediation MR analysis, we discovered a causal relationship between the levels of T-cell surface glycoprotein CD5 and DD, with a minor portion of this effect being mediated through *g_Bilophila* and the biosynthesis pathway of inosine 5’-phosphate I. This research holds significant implications for the exploration of the pathogenesis and prevention of DD.

## Author contributions

**Conceptualization:** Jie Zhou.

**Data curation:** Jie Zhou.

**Formal analysis:** Jie Zhou, Haitao Wang.

**Funding acquisition:** Yixin Xu.

**Investigation:** Jie Zhou, Yixin Xu, Haitao Wang, Kun Wang, Chao Chen.

**Methodology:** Jie Zhou, Yixin Xu, Kun Wang, Chao Chen.

**Resources:** Jie Zhou.

**Software:** Jie Zhou.

**Supervision:** Yixin Xu, Haitao Wang.

**Writing – original draft:** Jie Zhou.

**Writing – review & editing:** Yixin Xu.

## Supplementary Material




