# Supplementary material for: Investigating the causal links among gut microbiome features, inflammation-related proteins, and diverticular disease: Insights from a mediation Mendelian randomization study
Source: Medicine (Baltimore). 2025 May 30;104(22):e42676. doi: 10.1097/MD.0000000000042676 (PMC12129536; doi:10.1097/MD.0000000000042676)
Supplement: Supplementary file 2 [file medi-104-e42676-s002.pdf]

Supplementary Figure S1: Leave-One-Out Sensitivity Analysis: Assessing the Mediating Role of *g_Bilophila* in the Causal Pathway Linking T-Cell Surface Glycoprotein CD5 Levels to DD.

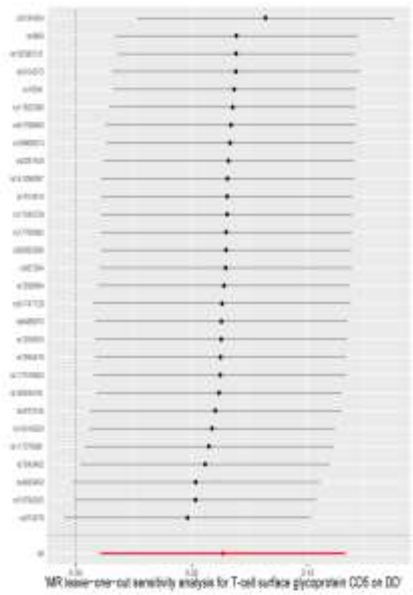

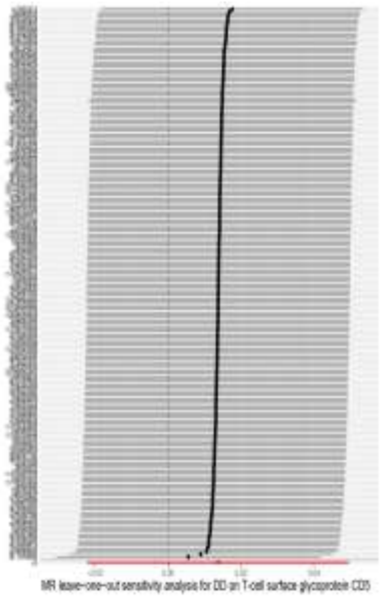

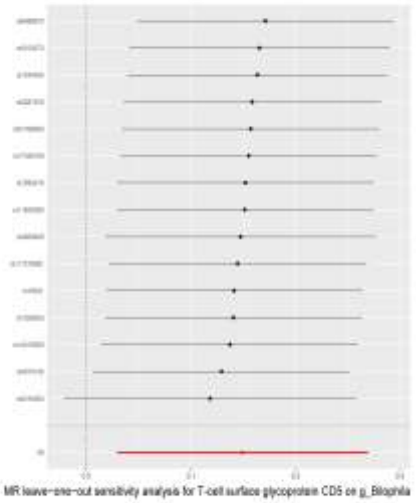

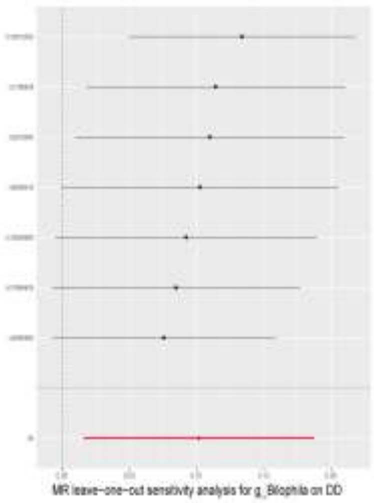

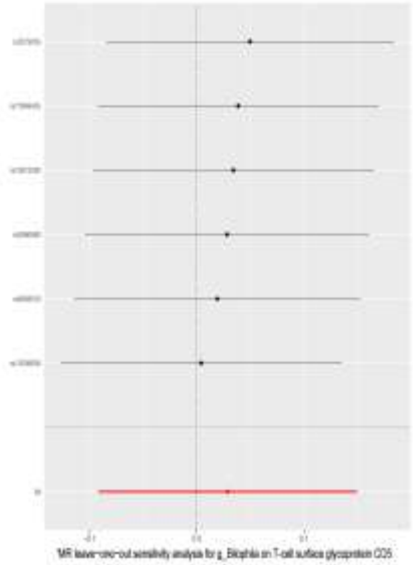

Supplementary Figure S1: Leave-One-Out Sensitivity Analysis: Assessing the Mediating Role of g_Bilophila in the Causal Pathway Linking T-Cell Surface Glycoprotein CD5 Levels to DD. DD, Diverticular Disease; MR, Mendelian Randomization.

Supplementary Figure S2: Leave-One-Out Sensitivity Analysis: Assessing the Mediating Role of Inosine 5'-phosphate Biosynthesis I Pathway in the Causal Pathway Linking T-Cell Surface Glycoprotein CD5 Levels to DD.

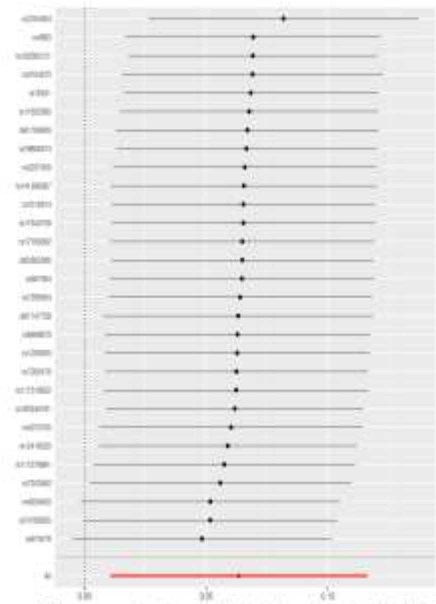

MR leave−one−out sensitivity analysis for T-cell surface glycoprotein CD5 on DD

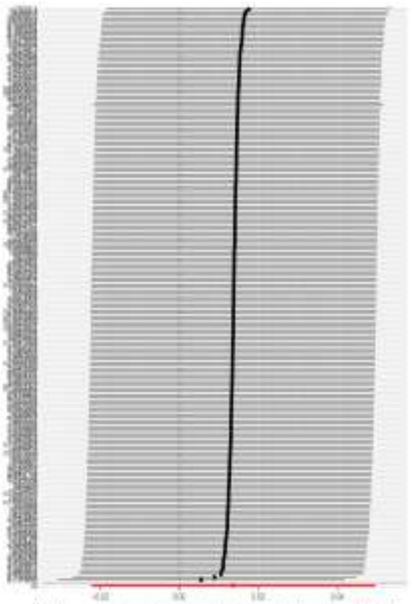

MR leave−one−out sensitivity analysis for DD on T-cell surface glycoprotein CD5

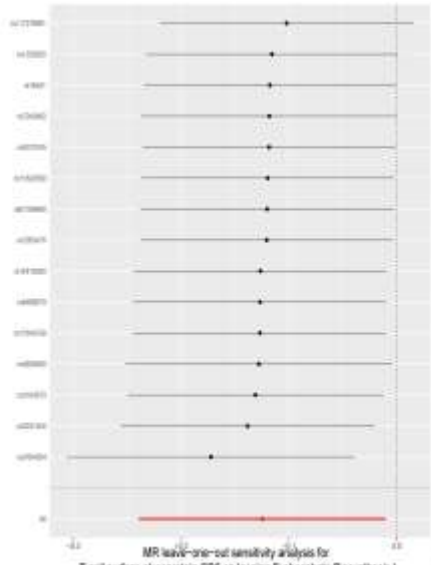

MR leave−one−out sensitivity analysis for
T-cell surface glycoprotein CD5 on Inosine 5'-phosphate Biosynthesis I

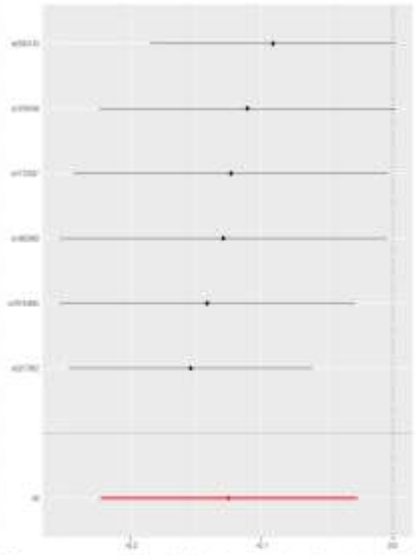

MR leave−one−out sensitivity analysis for Inosine 5'-phosphate Biosynthesis I on DD

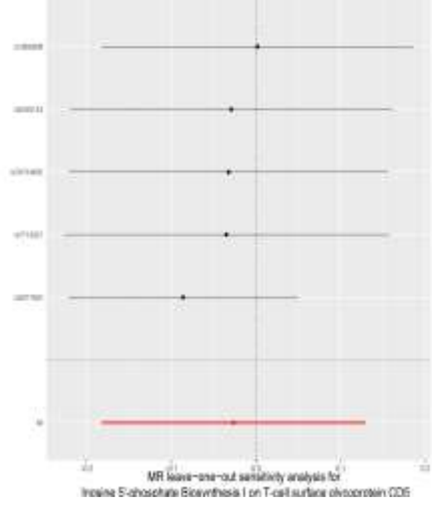

MR leave−one−out sensitivity analysis for
Inosine 5'-phosphate Biosynthesis I on T-cell surface glycoprotein CD5

Leave-One-Out Sensitivity Analysis: Assessing the Mediating Role of Inosine 5'-phosphate Biosynthesis I Pathway in the Causal Pathway Linking T-Cell Surface Glycoprotein CD5 Levels to DD. DD, diverticular disease; MR, Mendelian Randomization.
